# Thrombocytopenia and anemia associated with piperacillin-tazobactam: integrating clinical case series with FAERS disproportionality analysis

**DOI:** 10.3389/fphar.2026.1817103

**Published:** 2026-05-21

**Authors:** Jing Lu, Guoshang Yang, Shengjun Liang, Qifeng Yang, Changchun Xie, Wei Gao

**Affiliations:** 1 Pharmacy Department, Liwan Central Hospital of Guangzhou, Guangzhou, China; 2 Department of Urology, Liwan Central Hospital of Guangzhou, Guangzhou, China; 3 Endocrinology and Metabolism Department, Liwan Central Hospital of Guangzhou, Guangzhou, China

**Keywords:** adverse drug reaction, drug-associated anemia, drug-associated thrombocytopenia, FAERS database, pharmacovigilance, piperacillin

## Abstract

**Background:**

Piperacillin-tazobactam (TZP) is a commonly used broad-spectrum antibiotic in clinical practice; however, its haematopoietic toxicity profile has not yet been fully elucidated. This study combines clinical case observations with large-scale pharmacovigilance data to characterize TZP-associated thrombocytopenia and anemia.

**Methods:**

We first report a case series of three patients presenting with distinct TZP-associated haematopoietic injuries, including severe thrombocytopenia complicated by mild anemia, as well as mixed haematopoietic toxicity predominantly manifested as anemia, with or without concomitant thrombocytopenia. Subsequently, a disproportionality analysis was performed on the FDA Adverse Event Reporting System (FAERS) database using four quantitative methods: Reporting Odds Ratio (ROR), Proportional Reporting Ratio (PRR), Bayesian Confidence Propagation Neural Network (BCPNN), and Multi-item Gamma Poisson Shrinker (MGPS).

**Results:**

The disproportionality analysis confirmed a significant and robust risk signal for TZP-associated thrombocytopenia (ROR = 5.26, 95% CI 4.81–5.74), which remained stable in sensitivity analyses adjusting for concomitant medications. Although the signal for anemia was relatively weak across all methods, the clinical cases provided key evidence supporting the occurrence of this adverse reaction.

**Conclusion:**

TZP can induce severe thrombocytopenia. It is recommended to actively monitor complete blood counts, with particular attention to platelet, erythrocyte, and hemoglobin levels, within the first week of treatment initiation. When encountering unexplained cytopenia in clinical practice, TZP should be considered as a potential causative agent; if the drug is highly suspected, immediate discontinuation is warranted.

## Introduction

Piperacillin, commonly used in combination with β-lactamase inhibitors (e.g., tazobactam), is a broad-spectrum antibiotic and a first-line empiric therapy for moderate to severe infections in hospitalized patients due to its efficacy and generally favorable tolerability profile (Young and Plosker, 2001; Gin et al., 2007). Despite the widespread clinical use of piperacillin-tazobactam (TZP), the risk of haematopoietic toxicity and its potentially serious consequences cannot be overlooked (Wang et al., 2020; Gerhardy et al., 2021).

The clinical significance of this adverse effect is heightened by its potential severity and diagnostic complexity. According to prescribing information and literature reports, TZP-associated haematopoietic adverse reactions are relatively rare (<1%) (ElSalem et al., 2019). Mechanistically, TZP-associated cytopenias involve diverse immune and non-immune mechanisms (Tomar et al., 2012; Lee et al., 2009). This leads to heterogeneous presentations including isolated thrombocytopenia, anemia (e.g., immune hemolytic anemia), or a mixed picture. This mechanistic diversity contributes to key diagnostic challenges: an insidious onset often obscured by the underlying infection, potential for rapid progression, and a current reliance on anecdotal case reports due to a lack of systematic, large-scale epidemiological data.

To address these evidence gaps, pharmacovigilance databases such as the U.S. FDA Adverse Event Reporting System (FAERS) are invaluable. Through disproportionality analysis, these repositories can transform sporadic clinical observations into quantifiable risk signals, enabling the detection of rare adverse drug reactions (Morris et al., 2024). However, database analyses alone often lack detailed clinical context.

Therefore, this study employs a convergent design to provide a more comprehensive risk assessment. First, we present a detailed clinical case series to delineate the varied phenotypes of TZP-associated hematotoxicity. Second, we perform a comprehensive disproportionality analysis of the FAERS database to quantify and validate population-level signals for thrombocytopenia and anemia associated with TZP, with specific attention to high-risk demographics. By integrating in-depth clinical characterization with large-scale epidemiological analytics, this research aims to improve the recognition and risk stratification of this complication and to inform evidence-based monitoring guidelines.

## Case descriptions

### Case 1: positive drug rechallenge with severe thrombocytopenia and mild anemia

An 89-year-old male with a history of bladder malignancy (stage cT1N0M0), benign prostatic hyperplasia, and hydronephrosis was admitted for hematuria. On admission, vital signs were as follows: temperature 36.6 °C, respiratory rate 18 breaths/min, heart rate 79 beats/min, and blood pressure 147/69 mmHg. The patient had a history of hypertension, for which he regularly took valsartan-hydrochlorothiazide tablets, and denied history of chronic conditions such as coronary heart disease or diabetes mellitus. He underwent right nephrolithotomy several years ago. On 18 July 2025, all baseline laboratory parameters at admission were within normal ranges (platelet count: 182 × 10^9^/L; hemoglobin: 123 g/L; red blood cell count: 4.05 × 10^12^/L; white blood cell count: 8.68 × 10^9^/L; neutrophil percentage: 58.2%). Ultrasound indicated left hydronephrosis (to be differentiated from renal cyst) and benign prostatic hyperplasia. Chest CT showed signs of chronic bronchitis and emphysema in both lungs. Concomitant medications included tamsulosin hydrochloride sustained-release capsules and valsartan-hydrochlorothiazide tablets. Intravenous TZP was initiated on the same day (Day 1) at a dosage of 2.25 g every 12 h for perioperative antimicrobial prophylaxis.

On July 22, the patient underwent transurethral resection of bladder lesion under combined spinal and epidural anesthesia (CSEA). The procedure was uneventful, with a duration of 1 h and 50 min and an intraoperative blood loss of 20 mL. No postoperative discomfort was reported by the patient. Postoperative laboratory tests revealed a platelet count of 65 × 10^9^/L, hemoglobin of 107 g/L, red blood cell count of 3.56 × 10^12^/L, white blood cell count of 8.40 × 10^9^/L and neutrophil percentage of 71.1%. Inflammatory markers were as follows: C-reactive protein (CRP) 4.51 mg/L and procalcitonin (PCT) 0.039 ng/mL.

**FIGURE 1:**
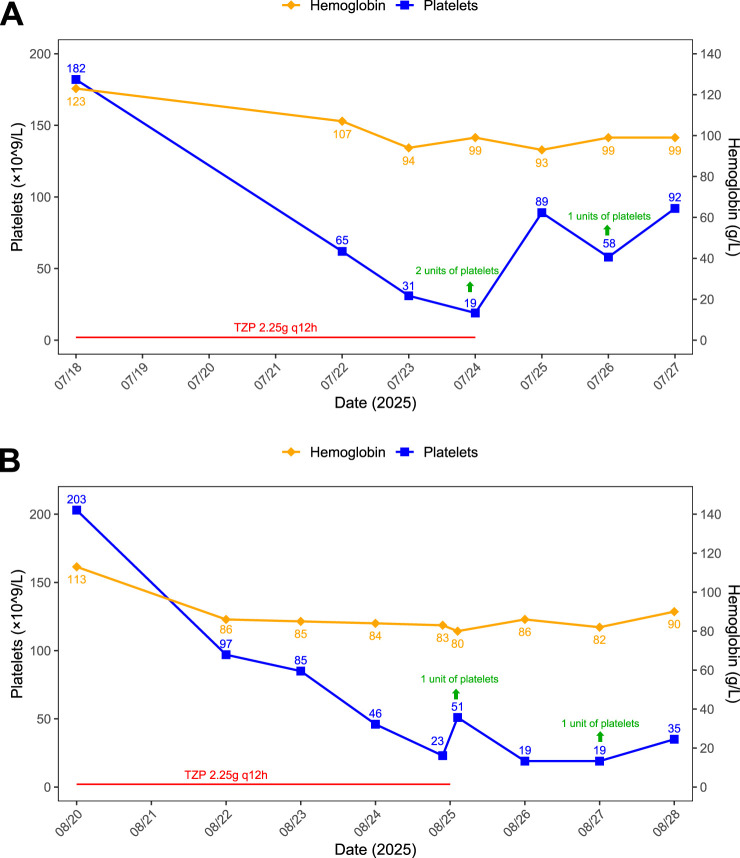
Trends in hemoglobin and platelet counts during the hospitalizations of Case 1. **(A)** First hospitalization. **(B)** Second hospitalization.

The platelet count dropped to its nadir of 19 × 10^9^/L on July 24 (Day 6), accompanied by a mild decrease in hemoglobin level (97 g/L). The platelet count recovered following the transfusion of 2 units of platelets on July 24, but it declined again to a nadir of 58 × 10^9^/L on July 26 (Day 8), which prompted the transfusion of 1 unit of platelets. A reexamination on July 27 (Day 9) showed a platelet count of 92 × 10^9^/L. TZP was discontinued on July 24. The patient was discharged in generally good condition, and no specific discomfort, including bleeding-related symptoms such as hematuria, was noted during the thrombocytopenic period. Details are presented in Figure 1A.

Since the first platelet test after admission on July 22 was performed immediately after the urological surgery, the initial clinical suspicion was that the thrombocytopenia might be postoperative in nature. After the onset of thrombocytopenia, relevant laboratory tests were conducted for the patient, with the results showing negative for dengue virus antigen and antibodies (IgG, IgM) as well as anticardiolipin antibodies (IgG, IgM, IgA). The quantitative level of anti-double stranded DNA antibody was 2.3 IU/mL (reference range: 0–24 IU/mL) and antinuclear antibody (ANA) titer was <1:100 (reference range: <1:100). The levels of immunoglobulins were as follows: IgG 16.19 g/L (reference range: 8.6–17.4 g/L), IgA 2.47 g/L (reference range: 1–4.2 g/L) and IgM 0.54 g/L (reference range: 0.3–2.2 g/L). Complement levels were C3 1.15 g/L (reference range: 0.7–1.4 g/L) and C4 0.24 g/L (reference range: 0.1–0.4 g/L).

On 20 August 2025, approximately 1 month after the previous admission, the patient was readmitted for transurethral resection of the prostate. At this admission, vital signs were as follows: temperature 36.6 °C, respiratory rate 22 breaths/min, heart rate 78 beats/min, and blood pressure 132/69 mmHg. Laboratory findings included hemoglobin 113 g/L, red blood cell count 3.81 × 10^12^/L, white blood cell count 7.62 × 10^9^/L, neutrophil percentage 57.0%, and platelet count 203 × 10^9^/L (within normal range). Concomitant medications included tamsulosin hydrochloride sustained-release capsules, valsartan-hydrochlorothiazide tablets, montelukast sodium tablets, and compound methoxyphenamine capsules. Upon readmission, TZP (2.25 g every 12 h) was reinitiated because the patient had symptoms of a lower respiratory tract infection and the thrombocytopenia observed during the prior hospitalization had not been attributed to TZP.

On August 22, the patient underwent transurethral resection of the prostate under CSEA. The procedure was uneventful, with a duration of 1 h and 25 min and intraoperative blood loss of 30 mL. The patient reported no postoperative discomfort. Postoperative inflammatory markers were as follows: CRP 3.11 mg/L, PCT 0.02 ng/mL. Within days of re-exposure, the patient developed a progressive decline in platelet count, experiencing a recurrence of severe thrombocytopenia nearly identical to the prior episode, accompanied by anemia (hemoglobin 86 g/L). The count dropped to 23 × 10^9^/L by August 25. Following a multidisciplinary consultation, the toxicity was attributed to TZP. The drug was discontinued immediately upon diagnosis, and after a transfusion of 1 unit of platelets, the count rose to 51 × 10^9^/L. However, on August 26 and 27, subsequent monitoring revealed a decline again to 19 × 10^9^/L. Another transfusion of 1 unit of platelets raised the count to 35 × 10^9^/L, after which the condition stabilized. The patient was discharged on August 28 with oral thrombopoietic agents prescribed. Details are presented in Figure 1B.

Causality Assessment: The positive rechallenge with a nearly identical haematopoietical profile provides strong clinical evidence for a causal association (Naranjo score: 10, indicating definite). The individual scores of the Naranjo scale are shown in Supplementary Table S1.

### Case 2: severe anemia accompanied by mild thrombocytopenia

A 54-year-old female with type 2 diabetes was admitted on 13 October 2025, for a diabetic foot infection complicated by ketoacidosis. She had a 3-year history of elevated blood glucose and presented with right foot ulceration for 1 week. She had a 3-year history of hypertension but did not take medication regularly, and denied history of coronary heart disease or other chronic conditions. On admission, vital signs were as follows: temperature 36.3 °C, respiratory rate 20 breaths/min, heart rate 101 beats/min, and blood pressure 112/71 mmHg. Laboratory findings included white blood cell count 25.93 × 10^9^/L, neutrophil percentage 89.3%, red blood cell count 3.15 × 10^12^/L, CRP 125.6 mg/L, PCT 1.11 ng/mL, unconjugated bilirubin 38.8 μmol/L, total bilirubin 39.5 μmol/L, and blood ketone 1.2 mmol/L. Her baseline hemoglobin level at presentation was 92 g/L. Intravenous TZP (4.5 g every 8 h) was initiated after admission. Concomitant medications included insulin aspart injection, lipoic acid injection, moxifloxacin sodium chloride injection, and nifedipine controlled-release tablets.

**FIGURE 2:**
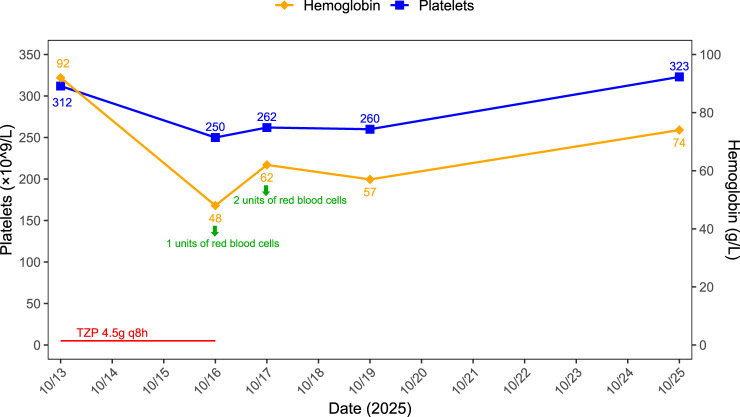
Trends in hemoglobin and platelet counts during the hospitalization of Case 2.

On October 16 (Day 3), repeat laboratory tests showed white blood cell count 23.99 × 10^9^/L, red blood cell count 1.63 × 10^12^/L, neutrophil percentage 85.3%, CRP 224.83 mg/L, PCT 0.913 ng/mL, unconjugated bilirubin 52.6 μmol/L, total bilirubin 86.3 μmol/L, and blood ketone 1.2 mmol/L. On the same day, the patient’s hemoglobin level dropped sharply to 48 g/L, representing a 48% decrease from baseline. Due to inadequate clinical response despite decreasing PCT levels, the anti-infective therapy was switched from TZP to imipenem-cilastatin on October 16. A total of 3 units of packed red blood cells were transfused on October 16 and 17. Subsequently, by October 19 (Day 6), the hemoglobin level had risen to 57 g/L, total bilirubin had decreased to 28.4 μmol/L, and unconjugated bilirubin was 16.9 μmol/L. By October 25, the hemoglobin level had further increased to 74 g/L. The patient recovered well and was discharged. A follow-up complete blood count on November 20 showed that her hemoglobin had returned to 118 g/L. In addition, her bilirubin levels had normalized, with unconjugated bilirubin at 8.0 μmol/L and total bilirubin at 13.0 μmol/L. Although a mild downward trend in platelet count was noted during TZP therapy, it remained within normal limits. Details are presented in Figure 2. Following the change in antibiotics and transfusion support, the hemoglobin level steadily recovered.

Causality Assessment: The close temporal relationship between drug initiation and the acute, severe drop in hemoglobin, coupled with recovery after discontinuation, supports a probable association (Naranjo score: 6, Supplementary Table S1).

### Case 3: isolated drug-associated anemia

A 94-year-old female was admitted on 31 December 2025, for a urinary tract infection. She presented with altered mental status and lethargy for 1 day, and was diagnosed with urinary tract infection, possibly sepsis. She had a history of hyperthyroidism, with subsequent hypothyroidism following radiation therapy many years ago, for which she took levothyroxine 0.1 mg daily. She had no history of coronary heart disease, hypertension, or other chronic conditions. On admission, vital signs were as follows: temperature 36.6 °C, respiratory rate 20 breaths/min, heart rate 86 beats/min, and blood pressure 115/86 mmHg. Laboratory findings included white blood cell count 13.53 × 10^9^/L, neutrophil percentage 77.9%, red blood cell count 3.54 × 10^12^/L, PCT 2.84 ng/mL (CRP was not tested), unconjugated bilirubin 4.1 μmol/L, total bilirubin 5.9 μmol/L. Her baseline hemoglobin at admission was 111 g/L. She received empirical therapy with cefotetan for 4 days. A follow-up complete blood count on 4 January 2026, showed a hemoglobin level of 108 g/L, with no significant change from baseline. Concomitant medications included levothyroxine, potassium chloride extended-release tablets, sodium bicarbonate tablets, and alprazolam tablets.

On 4 January 2026, based on the patient’s blood culture results, which revealed *Escherichia coli* resistant to second-generation cephalosporins and susceptible to TZP, the antimicrobial therapy was switched from cefotetan to intravenous TZP (2.25 g every 8 h). Within 3 days of initiating TZP, the hemoglobin level dropped to 85 g/L on January 7 in the absence of bleeding. On the same day, repeat laboratory tests showed white blood cell count 11.20 × 10^9^/L, neutrophil percentage 71.4%, red blood cell count 2.73 × 10^12^/L, and PCT 0.25 ng/mL, unconjugated bilirubin 3.9 μmol/L, total bilirubin 12.3 μmol/L. The patient’s clinical symptoms and inflammatory markers improved, indicating that the infection was effectively controlled, despite a marked decrease in red blood cell count and hemoglobin levels.

**FIGURE 3:**
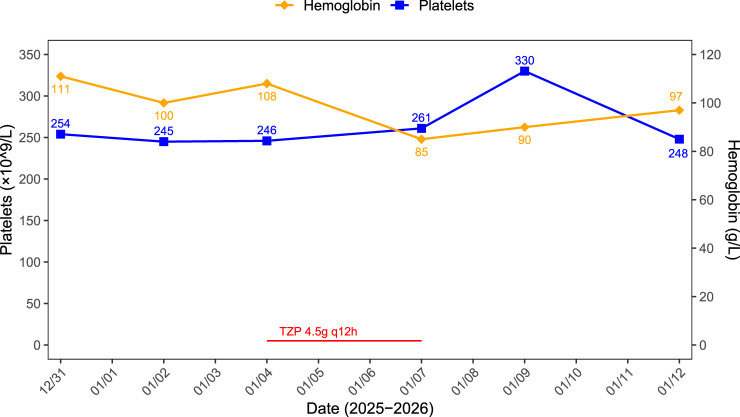
Trends in hemoglobin and platelet counts during the hospitalization of Case 3.

Following consultation with a clinical pharmacist, TZP-associated anemia was considered a possibility and could not be ruled out. Consequently, TZP was discontinued on 7 January 2026, and antimicrobial therapy was switched to meropenem. Subsequently, the hemoglobin level began to rise without the need for transfusion, reaching 97 g/L by January 12. The patient recovered well and was discharged. Details are presented in Figure 3.

Causality Assessment: The hematological decline occurred specifically after the introduction of TZP, with stabilization on the prior antibiotic and recovery upon its discontinuation. This pattern supports a probable association (Naranjo score: 7, Supplementary Table S1).

## FAERS database analysis results

### Descriptive results for the population

Following FDA-recommended data cleaning rules, a total of 9,066 TZP-related reports were identified from Q1 2004 to Q2 2025, involving 27,508 adverse drug events. Using the narrow Standardized Medical Queries (SMQs) for “Haematopoietic thrombocytopenia” (SMQ code: 20000031) and “Haematopoietic erythropenia” (SMQ code: 20000029), 501 reports of TZP-associated thrombocytopenia and 26 reports of TZP-associated anemia were identified. Details are presented in Supplementary Figure S1.

For thrombocytopenia, a male predominance was observed (58.3%). The median age of the patients was 63 years (interquartile range: 48–77). Notably, the age group of 65 years or older comprised the largest proportion (42.9%), followed by the 45–64 years group (26.3%). A total of 87.6% of the reports were submitted by health professionals (pharmacist, physician, other health professional).

For anemia, the gender distribution was balanced (53.8% male). The median age of the patients was 63 years (interquartile range: 46–76), with the elderly (≥65 years) representing the most affected age group (46.2%). A total of 73.1% of the reports were submitted by health professionals (pharmacist, physician, other health professional). Details are presented in Table 1.

**TABLE 1:** Characteristics of reports on piperacillin-tazobactam in the FAERS database.

Characteristics	Haematopoietic thrombocytopenia		Haematopoietic erythropenia	
	Cases	Proportion	Cases	Proportion
Sex, n (%)				
Female (%)	177	35.3	12	46.2
Male (%)	292	58.3	14	53.8
Not specified (%)	32	6.4	0	0
Age, years, n (%)				
<18 (%)	23	4.6	1	3.8
18–44 (%)	73	14.6	5	19.2
45–64 (%)	132	26.3	7	26.9
≥65 (%)	215	42.9	12	46.2
Median (Q1, Q3)	63 (48, 77)	​	63 (46, 76)	​
Not specified (%)	58	11.6	1	3.8
Reporters, n (%)				
Consumer (%)	36	7.2	4	15.4
Not specified (%)	26	5.2	3	11.5
Other health-professional (%)	114	22.8	3	11.5
Pharmacist (%)	200	39.9	6	23.1
Physician (%)	125	25.0	10	38.5

### FAERS signal detection results

The disproportionality analysis revealed a significant and robust signal for TZP-associated thrombocytopenia based on the SMQ definition of Haematopoietic thrombocytopenia. All four algorithms exceeded their respective signal detection thresholds: Reporting Odds Ratio (ROR) = 5.26 (95% CI 4.81–5.74), Proportional Reporting Ratio (PRR) = 5.18 (χ^2^ = 1691.44), Information Component (IC) = 2.37 (IC025 2.23), and Empirical Bayes Geometric Mean (EBGM) = 5.17 (EBGM05 4.73). These concordant results confirm a strong pharmacovigilance signal, and the findings were highly consistent across methods.

In contrast, the adverse reaction signals for Haematopoietic erythropenia under the SMQ were inconsistent and fell below the predefined thresholds. Although the point estimates for ROR and PRR were both 1.37 (greater than 1), the associated statistics did not meet the thresholds for a positive signal: the χ^2^ value for the PRR was low (2.57), and the IC025 (−0.12) and EBGM05 (0.93) values were below their critical limits. These findings suggest that the signal for TZP-associated anemia in the FAERS database remains unclear, with a weak clinical association. Details are presented in Table 2.

**TABLE 2:** Shows the results of the four analysis methods for the two SMQs.

SMQ	ROR (95% CI)	PRR (χ^2^)	IC (IC025)	EBGM (EBGM05)
Haematopoietic thrombocytopenia	5.26 (4.81–5.74)	5.18 (1691.44)	2.37 (2.23)	5.17 (4.73)
Haematopoietic erythropenia	1.37 (0.93 –2.01)	1.37 (2.57)	0.45 (−0.12)	1.37 (0.93)

SMQs: standardized medical queries; ROR: reporting odds ratio; PRR: proportional reporting ratio; χ^2^, chi-squared; IC, information component; IC025, IC025, the lower limit of 95% CI, of the IC; EBGM, empirical Bayesian geometric mean; EBGM05, the lower limit of 95% CI, of EBGM.

### Sensitivity analysis

An analysis of concomitant medications in 501 reports of Haematopoietic thrombocytopenia under the SMQ identified a total of 505 drugs, with an average of 3.8 drugs per patient. The names and frequencies of these concomitant medications are shown in Supplementary Table S2. We excluded 150 reports that involved nine drugs with well-established evidence of causing thrombocytopenia: vancomycin, heparin, enoxaparin, linezolid, dalteparin, carboplatin, quinine, cisplatin, and nadroparin. After exclusion, the four signal detection measures for Haematopoietic thrombocytopenia were as follows: ROR = 3.68 (95% CI 3.31–4.09), PRR = 3.65 (χ^2^ = 676.09), IC = 1.85 (IC025 1.70), and EBGM = 3.64 (EBGM05 3.28). The signal strength remained consistent with previous analyses, supporting the robustness of the data and the reliability of the conclusions in this study.

## Discussion

In this study, we combined detailed clinical case descriptions with a large-scale FAERS database disproportionality analysis to characterize TZP-associated haematopoietic toxicity. The key findings are threefold. First, the three presented cases illustrate distinct clinical phenotypes: severe thrombocytopenia with mild anemia (positive rechallenge), severe anemia with mild thrombocytopenia, and isolated anemia. Second, the FAERS disproportionality analysis revealed a strong, robust signal for TZP-associated thrombocytopenia, which remained stable after excluding reports with concomitant thrombocytopenic drugs. Third, in contrast, the signal for TZP-associated anemia was weak and did not meet predefined thresholds across all four detection methods, despite the occurrence of clinically significant anemia in our case series.

Since TZP is an anti-infective agent and haematopoietic abnormalities may also arise from the progression of infection itself, it is challenging to determine the causal relationship between TZP and haematopoietic toxicity. Our findings are consistent with previous pharmacovigilance studies, all confirming a significant association between TZP and thrombocytopenia (Aster and Bougie, 2007; Fritz et al., 2025). However, for TZP-associated anemia, the existing literature is limited and conclusions remain inconsistent: although case reports have suggested that TZP can induce immune hemolytic anemia (Meinus et al., 2016; Mayer et al., 2015), systematic studies are lacking, which aligns with the findings of the present study. Although individual cases support an association between TZP and anemia, the disproportionality analysis based on the large-scale FAERS database showed a weak signal.

This apparent contradiction between “clinically observable” anemia and “weak population signal” prompted us to further explore the relationship between TZP and anemia. In routine clinical practice, when a patient with an infectious disease (especially severe infection) experiences a drop in hemoglobin, clinicians typically attribute it to the infection itself (Denstaedt et al., 2023). Indeed, anemia is extremely common in critically ill patients: a study of ICU patients (excluding those with end-stage renal disease or primary haematopoietic disorders) showed that 77% developed anemia during hospitalization (Von Ahsen et al., 1999). The mechanisms include blood loss (repeated phlebotomy, gastrointestinal bleeding, surgery) and anemia of inflammation (shortened red blood cell survival, dysregulated iron metabolism, bone marrow suppression, etc.) (Aird, 2003; Loftus et al., 2019). Consequently, in Cases 1 and 2 of this study, anemia was not initially attributed to TZP: the hemoglobin decline in Case 1 was thought to be related to surgery, while that in Case 2 was attributed to the infection itself. Only after discussions between the clinical pharmacist and the physicians, when the infection was inadequately controlled, TZP was discontinued, and the antibiotic was upgraded to imipenem, the possibility of TZP-associated anemia was recognized. What truly convinced us that TZP can cause anemia was Case 3. Having undergone multidisciplinary discussions in the previous two cases, the pharmacist was already involved in monitoring anti-infective efficacy when the patient was switched from cefotetan to TZP. Therefore, when a drop in hemoglobin was detected on repeat testing 3 days after TZP initiation, we promptly discontinued the drug, and the patient’s hemoglobin gradually recovered without the need for transfusion. This dechallenge process provides strong evidence for TZP-associated anemia.

In summary, despite the weak signal from the large-scale FAERS database, our case series suggests that TZP can indeed cause clinically significant anemia in some patients. This discrepancy between “weak signal and strong clinical evidence” may be explained by the following reasons: (1) the high background noise of anemia in infected patients dilutes the drug signal; (2) clinicians often attribute anemia to underlying diseases, leading to underreporting of TZP-associated anemia; (3) drug-associated anemia may involve individual susceptibility (e.g., immune-mediated mechanisms) and does not occur universally. Therefore, for an unexplained drop in hemoglobin during TZP therapy, clinical vigilance should be maintained even if the database signal is weak.

At present, the hematological toxicity of TZP is considered to be mainly mediated by two pathways: immune-mediated mechanism and direct toxic mechanism. The immune-mediated pathway is the most extensively studied and clinically relevant mechanism, characterized by the formation of drug-dependent antibodies (DDAbs) that target platelets and red blood cells. TZP, as a hapten, binds to platelet membrane proteins or red blood cell surface antigens, forming complete antigens that stimulate the immune system to produce specific IgG antibodies (Aster and Bougie, 2007). These antibodies recognize and bind to drug-platelet or drug-red blood cell complexes, leading to complement activation and subsequent cell destruction. In the case of thrombocytopenia, DDAbs primarily target glycoproteins on the platelet membrane, resulting in platelet clearance by macrophages in the spleen and liver (Vayne et al., 2020; Loo et al., 2012). For anemia, drug-induced antibodies react with red blood cell membrane antigens, activating the complement system and causing intravascular hemolysis (Meinus et al., 2016; Mayer et al., 2015). Direct toxic effects of piperacillin on hematopoietic cells are less common but can occur with prolonged or high-dose therapy (Lee et al., 2009). The drug may directly damage bone marrow precursors, leading to decreased production of platelets and red blood cells (Xia et al., 2025). It is important to note that the mechanism of direct toxicity is often associated with the cumulative dose of TZP. Reduced drug metabolism and excretion capacity can easily lead to drug accumulation in the body, increasing the risk of direct hematotoxicity. This risk is particularly relevant in elderly patients, who are generally considered to have diminished drug metabolism and excretion capacity (Fu et al., 2024).

This study has certain limitations that need to be objectively explained. First, the sample size of this study is limited. The number of patients with TZP-associated thrombocytopenia and anemia included is relatively small, especially the number of patients with anemia (n = 26), which may lead to bias in the analysis of risk factors (such as the influence of combined medication and liver and kidney function) and reduce the statistical power of the study. Secondly, this study employed a retrospective case analysis combined with a disproportionality analysis of a spontaneous reporting database and thus lacked prospective validation. The conclusions drawn (such as risk factors for hematotoxicity) still require further confirmation through large-scale prospective clinical studies.

## Conclusion

TZP is associated with a strong and robust pharmacovigilance signal for thrombocytopenia, which remains stable after adjusting for concomitant medications. In contrast, the signal for TZP-associated anemia is weak in the FAERS database despite clear evidence of causality from clinical case series. This discrepancy likely reflects high background noise, underreporting, and individual susceptibility. Clinicians should maintain vigilance for both haematopoietic adverse events during TZP therapy, with particular attention to platelet, hemoglobin, and red blood cell levels within the first week of treatment. Unexplained cytopenias should prompt consideration of TZP as a potential cause, and timely discontinuation is warranted when drug-associated toxicity is suspected.

## Methods

This study combines two complementary components: (1) a single-center retrospective case series of patients with suspected TZP-associated hematologic adverse events, and (2) a pharmacovigilance disproportionality analysis using the FAERS database. The case series component was conducted at a 1,000-bed urban tertiary hospital in Guangzhou, China, with clinical data extracted retrospectively from electronic medical records. The pharmacovigilance component utilized reports submitted to the FAERS database from Q1 2004 through Q2 2025. All case details were de-identified prior to analysis.

### Data sources

The data for this study were sourced from adverse event reports in the FAERS database, covering Q1 2004 to Q2 2025. FAERS is an open-access database that tracks the safety profiles of drugs on the market. The FAERS database contains 7 structured datasets: DEMO (demographic characteristics), DRUG (drug exposure information), REAC (adverse reaction details), OUTC (clinical outcome), RPSR (report source), THER (duration of drug therapy), and INDI (treatment indication information). The FAERS data used in this study were collected, cleaned, and preprocessed using SAS software (version 9.4, SAS Institute Inc., Cary, NC, USA). This process included encoding and classifying the preferred terms (PTs) and Standardized MedDRA Queries (SMQs), which were recorded in the Medical Dictionary for Regulatory Activities (MedDRA, version 28.0).

### Data extraction and statistical analysis

Initially, we downloaded data pertaining to TZP, which included case ID, primary ID, indications, suspected drugs, adverse events, outcome events, reporter country, reporter type, sex, age, report date, start date, and event date. Following the FDA’s recommendations, we removed duplicate records from the “DEMO” table, retaining only one, and deleted the earliest “FDA_DT” column when the “CASEID” column was identical. We also removed the lesser “PRIMARYID” column when both the “CASEID” and “FDA_DT” columns matched. The disproportionality analysis was used to explore the potential association between TZP and adverse events, a standard approach in pharmacovigilance research, including Reporting Odds Ratio (ROR), Proportional Reporting Ratio (PRR), Bayesian Confidence Propagation Neural Network (BCPNN) and Multi-Item Gamma Poisson Shrinker (MGPS). Each method has distinct strengths. ROR excels in mitigating biases from small samples and is highly effective for early signal detection. PRR performs reliably with incomplete datasets. BCPNN boosts signal detection accuracy by integrating multi-source data and cross-validation, remaining powerful even with few reports. MGPS specializes in identifying rare event signals while minimizing random patterns. Combining these four complementary methods ensures more reliable and robust results, effectively offsetting the limitations of individual approaches. The summary of major algorithms used for signal detection is shown in Supplementary Tables S3,S4. Only signals that met the detection thresholds of all four algorithms were considered statistically significant in this study. Higher ROR values indicate stronger signal intensity, reflecting a robust statistical association between the drug and the adverse event.

### Sensitivity analysis

A review of the literature and the FAERS database revealed that other medications known to cause thrombocytopenia may be used concomitantly during treatment with TZP. Given the risk of drug-associated thrombocytopenia from concurrent medications, we excluded cases involving concomitant use of vancomycin, heparin, linezolid, low-molecular-weight heparins, platinum agents, or quinine, drugs with well-established evidence of causing thrombocytopenia. A sensitivity analysis was performed to confirm the robustness of the data.

## Data Availability

The original contributions presented in the study are included in the article/Supplementary Material, further inquiries can be directed to the corresponding author.
