# Supplementary material for: Thrombocytopenia and anemia associated with piperacillin-tazobactam: integrating clinical case series with FAERS disproportionality analysis
Source: Front Pharmacol. 2026 May 21;17:1817103. doi: 10.3389/fphar.2026.1817103 (PMC13233410; doi:10.3389/fphar.2026.1817103)
Supplement: Supplementary file 1 [file Supplementaryfile1.docx]

**
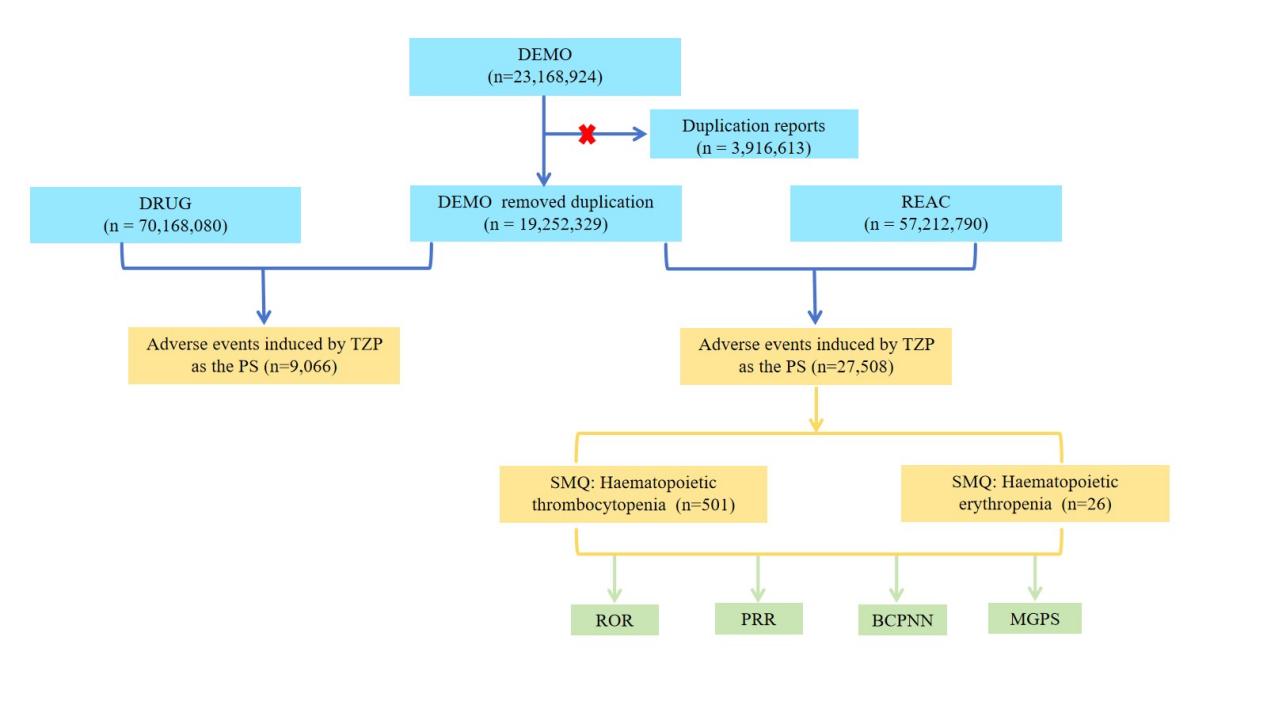
**

**Supplementary Figure 1.** **Flowchart for screening in FAERS database**

(DEMO, REAC, and DRUG are subsets of data from the FAERS database;DEMO: demographic characteristics; REAC: adverse reaction details; DRUG: drug exposure information; TZP: piperacillin-tazobactam; PS: primary suspected; SMQ:Standardized MedDRA Query; ROR: Reporting Odds Ratio; PRR: Proportional Reporting Ratio; BCPNN: Bayesian Confidence Propagation Neural Network; MGPS:Multi-item Gamma Poisson Shrinker. )

**Supplementary Table S1. Naranjo scores of adverse event**

| **Naranjo Adverse Drug Reaction Probability Scale** | **Yes** | **No** | **Do not know** | **Case 1** | **Case 2** | **Case 3** |
| --- | --- | --- | --- | --- | --- | --- |
| 1. Are there previous conclusive reports on this reaction? | +1 | 0 | 0 | +1 | +1 | +1 |
| 1. Did adverse event appear after the suspected drug was given? | +2 | -1 | 0 | +2 | +2 | +2 |
| 1. Did the adverse reaction improve when the drug was discontinued or a specific antagonist was given? | +1 | 0 | 0 | +1 | +1 | +1 |
| 1. Did the adverse reaction appear when the drug was readministered? | +2 | -1 | 0 | +2 | 0 | 0 |
| 1. Are there alternative causes that could have caused the reaction? | -1 | +2 | 0 | -1 | -1 | 0 |
| 1. Did the reaction reappear when a placebo was given? | -1 | +1 | 0 | +1 | +1 | +1 |
| 1. Was the drug detected in any body fluid in toxic concentrations? | +1 | 0 | 0 | 0 | 0 | 0 |
| 1. Was the reaction more severe when the dose was increased, or less severe when the dose was decreased? | +1 | 0 | 0 | +1 | +1 | +1 |
| 1. Did the patient have a similar reaction to the same or similar drugs in any previous exposure? | +1 | 0 | 0 | +1 | 0 | 0 |
| 1. Was the adverse event confirmed by any objective evidence? | +1 | 0 | 0 | +1 | +1 | +1 |
| Total score |  |  |  | 10 | 6 | 7 |

**Supplementary Table S3. Four-grid table of ratio imbalance method**

|  | Target AEs | Other AEs | Total |
| --- | --- | --- | --- |
| Target drug | a | b | a+b |
| Other drugs | c | d | c+d |
| Total | a+c | b+d | a+b+c+d |

**Supplementary Table S4. Four major algorithms used for signal detection**

| **Algorithms** | **Equation** | **Criteria** |
| --- | --- | --- |
| ROR | ROR=ad/bc | lower limit of 95% CI>1, a≥3 |
|  | 95%CI=e^ln(ROR)±1.96(1/a+1/b+1/c+1/d)^0.5^ |  |
| PRR | PRR=a(c+d)/c/(a+b) | PRR≥2, χ^2^≥4, a≥3 |
|  | χ^2^=[(ad-bc)^2](a+b+c+d)/[(a+b)(c+d)(a+c)(b+d)] |  |
| BCPNN | IC=log_2_a(a+b+c+d)(a+c)(a+b) | IC025>0 |
|  | 95%CI= E(IC) ± 2V(IC)^0.5 |  |
| MGPS | EBGM=a(a+b+c+d)/(a+c)/(a+b) | EBGM05>2 |
|  | 95%CI=e^ln(EBGM)±1.96(1/a+1/b+1/c+1/d)^0.5^ |  |

**Notes:**

a, number of reports containing both the target drug and target adverse drug reaction;

b, number of reports containing other adverse drug reaction of the target drug;

c, number of reports containing the target adverse drug reaction of other drugs;

d, number of reports containing other drugs and other adverse drug reactions;

95%CI, 95% confidence interval; χ^2^, chi-squared; IC, information component; IC025, the lower limit of 95% CI of the IC; E(IC), the IC expectations; V(IC), the variance of IC; EBGM, empirical Bayesian geometric mean; EBGM05, the lower limit of 95% CI of EBGM.

**Supplementary Table S5. Summary of Reported Cases**

| **case** | **Age/Sex** | **Indication** | **TZP Start** | **TZP Stop** | **PLT Baseline (×10⁹/L)** | **PLT Nadir (×10⁹/L)** | **PLT Discharge (×10⁹/L)** | **PLT Transfusion** | **Hb Baseline (g/L)** | **Hb Nadir (g/L)** | **Hb Discharge (g/L)** | **RBC Transfusion** |
| --- | --- | --- | --- | --- | --- | --- | --- | --- | --- | --- | --- | --- |
| 1 | 89/M | Hematuria | 2025-07-18 | 2025-07-24 | 182 | 19 | 92 | Yes (3 units) | 123 | 94 | 99 | No |
|  |  | Benign prostatic hyperplasia | 2025-08-20 | 2025-08-25 | 203 | 19 | 35 | Yes (2 units) | 113 | 80 | 90 | No |
| 2 | 54/F | Diabetic foot infection | 2025-10-13 | 2025-10-16 | 312 | 250 | 323 | No | 92 | 48 | 74 | Yes (3 units) |
| 3 | 94/F | UTI, sepsis | 2026-01-04 | 2026-01-07 | 254 | 245 | 248 | No | 111 | 85 | 97 | No |

**Notes:**

TZP: Piperacillin-tazobactam; PLT: Platelet count; Hb: Hemoglobin; RBC: Red blood cell; Baseline: Measurement before treatment initiation; Nadir: Lowest value recorded during treatment; Discharge: Measurement at hospital discharge; Transfusion: Receipt of corresponding blood product transfusion; UTI: Urinary Tract Infection.
